# Fecal Microbiota Transplantation in Patients with HBV Infection or Other Chronic Liver Diseases: Update on Current Knowledge and Future Perspectives

**DOI:** 10.3390/jcm10122605

**Published:** 2021-06-12

**Authors:** Mattia Paratore, Francesco Santopaolo, Giovanni Cammarota, Maurizio Pompili, Antonio Gasbarrini, Francesca Romana Ponziani

**Affiliations:** 1 Internal Medicine and Gastroenterology-Hepatology Unit, Fondazione Policlinico Universitario Agostino Gemelli IRCCS, 00168 Rome, Italy; santopaolofrancesco@gmail.com (F.S.); giovanni.cammarota@unicatt.it (G.C.); maurizio.pompili@unicatt.it (M.P.); antonio.gasbarrini@unicatt.it (A.G.); francesca.ponziani@gmail.com (F.R.P.); 2 Dipartimento Universitario di Medicina e Chirurgia Traslazionale, Università Cattolica del Sacro Cuore, 00168 Rome, Italy

**Keywords:** gut microbiota, gut-liver axis, liver disease, fecal microbiota transplantation

## Abstract

Liver disease and gut dysbiosis are strictly associated, and the pathophysiology of this bidirectional relationship has recently been the subject of several investigations. Growing evidence highlights the link between gut microbiota composition, impairment of the gut-liver axis, and the development or progression of liver disease. Therefore, the modulation of gut microbiota to maintain homeostasis of the gut-liver axis could represent a potential instrument to halt liver damage, modify the course of liver disease, and improve clinical outcomes. Among all the methods available to achieve this purpose, fecal microbiota transplantation (FMT) is one of the most promising, being able to directly reshape the recipient’s gut microbial communities. In this review, we report the main characteristics of gut dysbiosis and its pathogenetic consequences in cirrhotic patients, discussing the emerging data on the application of FMT for liver disease in different clinical settings.

## 1. Introduction

Liver cirrhosis represents the end stage of all chronic liver diseases, including chronic hepatitis B virus (HBV) infection, and is the hallmark of gut dysbiosis. Although the pathophysiology remains unclear, several factors may contribute to the modifications of the gut microbiota observed in cirrhotic patients, which is characterized by the reduction in beneficial bacteria and the relative increase in potentially pathogenic ones [1,2]. In particular, *Bacteroidetes*, Lachnospiraceae, Ruminococcaceae, and *Clostridium incertae sedis XIV* are reduced while Proteobacteria, Fusobacteria, *Clostridium cluster XI*, Enterobacteriaceae, Enterococcaceae, Lactobacillaceae, Alcaligenaceae and Streptococcaceae are overabundant in cirrhotic patients [3]. *Streptococcus* spp. and *Veillonella* spp., which are bacteria of oral origin, are also enriched [4]. This supports the hypothesis that alterations of the gastrointestinal system in patients with advanced liver disease contribute to the gut microbiota derangement [4,5]. Indeed, portal hypertension, reduced secretion of gastric acid, impaired gastrointestinal motility, and local and systemic immunological dysfunction are the most important factors in shaping the intestinal bacterial community in this setting [6]. Changes in the composition of bile acids secreted into the intestine as a consequence of liver dysfunction are also implied in the gut microbiota derangement and in the maintenance of liver damage [7,8]. Primary bile acids represent a metabolic substrate for intestinal bacteria, which in turn produce secondary bile acids that can act as a regulator of the gut microbiota itself. Thus, a primary alteration of the gut microbiota can lead to the alteration of the bile acids pool, with toxic effects on the liver [9].

The alteration of the gut microbiota causes intestinal inflammation and worsens the damage to the gut barrier; in addition, the translocation of bacterial fragments and their metabolites can trigger liver injury and systemic inflammation [10]. Several studies have associated changes in the gut microbiota with liver cirrhosis and its complications, in particular hepatic encephalopathy (HE) [1].

It is also possible that in some individuals dysbiosis could precede the onset of liver disease, and may represent the first hit in the development of hepatic injury.

Although it is difficult to clarify whether the chicken or egg came first, the role of the gut microbiota in the pathogenesis of liver disease is crucial and, therefore, its modulation by fecal microbiota transplantation (FMT) is a promising therapeutic and, perhaps, preemptive option for these patients. The aim of this review is to provide an update on the current knowledge regarding the efficacy and safety of FMT in patients with hepatic encephalopathy and with chronic HBV infection or other chronic liver diseases.

## 2. FMT for Hepatic Encephalopathy

In cirrhotic patients with HE, the gut microbiota composition is characterized by an increase in potentially pathogenic bacteria, mostly Alcaligenaceae, Porphyromonadaceae, Veillonellaceae, and Enterobacteriaceae, and by a decreased abundance of commensal ones, such as Lachonospiraceae, Ruminococcaceae, and *Blautia*, compared to patients with compensated cirrhosis [11,12,13]. These changes in the gut microbiota composition are correlated with the accumulation of bacteria-derived products, including ammonia, mercaptans, benzodiazepine-like substances, and indoles. After passing the blood-brain barrier, these molecules impair astrocyte function, inducing osmotic stress, oxidative stress, mitochondrial dysfunction, and a decrease in excitatory neurotransmission [14]. In addition, the translocation of bacteria and their fragments, endotoxin, and DNA triggers a systemic inflammatory response, which acts synergistically with bacterial metabolic products in the development and progression of cognitive impairment in patients with HE [15,16]. The prevalence of some bacterial families, such as Enterobacteriaceae and Streptococcaceae, is associated with hyperammonemia-related astrocyte dysfunction, while the overgrowth of other species, such as Porphyromonadaceae, is related to brain interstitial edema and neuronal dysfunction [17]. Less complex correlations between bacteria and phages have been observed in cirrhotic patients with HE compared to controls, and hospitalization was associated with enrichment of *Streptococcus*-linked phages [18]. These findings further support the role of ammonia-generating, urease producing bacteria such as *Streptococcus* in the pathogenesis of HE. Urease is essential in bacterial colonization and is a virulence factor of several pathogenic bacteria such as *Streptococcus* spp., *Helicobacter pylori*, *Klebsiella* spp., *Escherichia coli* [19]. Urease allows bacteria to survive in unfavorable environments and exerts direct and indirect (i.e., through ammonia) toxic effects not only on the brain, but also on several host tissues.

FMT can be a promising tool in association with the standard of care (lactulose, rifaximin) for the modulation of the gut microbiota in patients with HE.

In a small open-label randomized trial, 20 cirrhotic outpatients with recurrent HE were randomized in two arms: FMT preceded by 5 days of broad-spectrum antibiotics or the standard of care (lactulose and rifaximin) (Table 1) [20]. All donor materials, instilled by enema, came from a single individual that was selected based on machine learning techniques to identify the highest relative abundances of Lachnospiraceae and Ruminococcaceae among a universal stool donor bank. FMT showed excellent safety, with no FMT-related serious adverse events (SAEs) at day 150 from intervention. HE recurred in 50% of patients in the standard of care (SOC) group, but no recurrence was observed in those who received FMT; FMT was also associated with a lower rate of hospitalizations and a higher improvement in cognitive tests. This was paralleled by the increase in microbial alpha diversity as well as in the relative abundance of beneficial taxa (Lactobacillaceae and Bifidobacteriaceae) compared to the SOC arm. Safety and efficacy were still maintained at 1-year from the procedure, with significantly more episodes of hospitalizations and HE occurring in the SOC arm compared to the FMT one [21].

The same group of Authors reported on the safety and tolerability of FMT with oral capsules in patients with recurrent HE in a randomized, single-blind, placebo-controlled trial (Table 1) [22]. Using the same criteria of the previous study for donor selection, 20 patients were randomized 1:1 to receive 15 capsules of FMT or placebo. No FMT-related SAE was observed, and the rate of hospitalization was lower in the FMT compared to the control group. However, HE episodes recurrence rates were similar between groups, and only one of the cognitive tests performed (EncephalApp) showed a significant improvement after FMT. A positive modification in the stool and mucosal microbial composition was observed. Indeed, in stool samples a reduction in potentially pathogenic bacteria (Veillonellaceae and Sutterellaceae) was observed; furthermore, the analysis of duodenal biopsy revealed a drop in potentially pathogenic bacteria (Streptococaceae and Veillonellaceae) as well as an increase in beneficial ones (Ruminococcaceae and Bifidobacteriaceae) [21].

With the limitation of the small sample size of these two studies, a lower gastrointestinal route preceded by administration of broad-spectrum antibiotics seems to favor FMT efficacy, being more effective to prevent HE recurrence. Considering the absence of significant SAEs, and the limited therapeutic options for patients with recurrent HE, further studies with larger sample sizes should be encouraged to clarify the best procedure to maximize the efficacy of FMT in cirrhotic patients with HE.

## 3. FMT for Different Etiologies of Liver Disease

### 3.1. HBV

The gut-liver axis could influence the host immune response to HBV, the susceptibility to hepatocyte damage, and the progression of liver disease to a more advanced stage of fibrosis.

Studies have demonstrated that changes in the gut microbiota may already be underway in the early phase of chronic HBV infection [30,31] and are mainly characterized by the reduction in *Alistipes* and *Bacteroides* compared to healthy subjects. This alteration of the gut microbiota results in the reduced production of SCFA and antibacterial peptides, consequently compromising the intestinal barrier [31]. Instead, Zeng et al. demonstrated that in chronic HBV hepatitis there was a higher abundance of Bacteroidetes and a lower abundance of Firmicutes and Actinobacteria, with enhanced activation of LPS-related pathways and disruption of the intestinal barrier [30].

Chen et al. analyzed gut microbiota alterations in healthy subjects, patients with chronic HBV infection, chronic HBV hepatitis, and liver cirrhosis, respectively. The results showed a dynamic shift of the gut microbiota profile during the progression of the disease. In particular, a progressive reduction in alpha-diversity was observed, as well as an increase in *Fusobacteria*, *Klebsiella*, *Veillonella*, and *Haemophilus* abundance, and a decrease in *Dialister succinatiphilus* and *Alistipes onderdonkii* in the late stage of liver disease [32].

Indeed, if minimal alterations of the gut microbiota are reported in patients with chronic HBV infection, the worsening of liver disease is associated with the decrease in beneficial species, such as *Roseburia* and *Ruminococcus*, and the increase in other potentially pathogenic species, such as *Escherichia*, *Shigella*, *Klebsiella*, *Enterococcus* and *Veillonella*. In patients affected by HBV-related acute on chronic liver failure, *Enterococcus*, *Klebsiella*, *Lactobacillus*, *Veillonella*, and *Escherichia-Shigella* become dominant [33]. 

A recent study assessed the gut microbiota composition in a mouse model of chronic HBV hepatitis, before and after treatment with entecavir, reporting that antiviral treatment can reverse dysbiosis developed in HBV-infected mice [34].

The pro-inflammatory alterations caused by dysbiosis in patients with HBV infection may contribute to the pathogenesis of hepatocellular carcinoma (HCC). Indeed, *Bacteroides*, *Lachnospiraceae* incertae sedis, and *Clostridium XIVa* are enriched in HCC patients with chronic HBV hepatitis and a high tumor burden [35].

Little evidence exists regarding the efficacy of FMT in chronic viral B hepatitis (Table 1). Ren et al. reported a pilot trial, analyzing the efficacy of FMT in 5 chronic hepatitis B patients treated with long-term antiviral therapy and without HBeAg clearance or seroconversion, compared to 13 chronic hepatitis B patients, in the same condition, who did not receive FMT. In the FMT group there was a significant HBeAg decline, nevertheless none achieved seroconversion. In contrast, none in the control group showed a decrease in HBeAg (Table 1) [23].

Another pilot study investigated the efficacy of FMT in achieving HBeAg clearance, HBsAg clearance, and reduction in HBV DNA serum level in HBeAg positive patients with chronic HBV hepatitis treated with long-term antiviral therapy (Table 1) [24]. Two out of 12 patients who received FMT obtained HBeAg clearance against none in the control group. In both groups, none of the patients achieved HBsAg clearance. After six months, a reduction in HBV DNA serum level was noticed in the FMT arm of patients with positive DNA at baseline, while no decrease was reported in the control group.

### 3.2. Hepatitis C Virus (HCV)

Two hypotheses can explain how HCV infection can influence the gut-liver axis [36,37]. On one hand, liver damage due to HCV infection changes liver function and, indirectly, the gut microbiota. On the other hand, HCV infection of B-lymphocyte and the consequent alteration of IgA production can lead to dysbiosis, increased bacterial translocation, and activation of inflammatory pathways that worsen the entity of liver damage and its progression to extensive fibrosis and cirrhosis.

HCV infected patients have lower bacterial diversity compared to healthy controls, with a decrease in Clostridiales (butyrate-producing bacteria), Lachnospiraceae, and Ruminococcaceae (SCFAs producers) and an increase in *Lactobacillus* and *Streptococcus* [38]. A transient increase in *Bacteroides* and Enterobacteriaceae, associated with proinflammatory effects, has been observed in patients with low fibrosis and normal serum levels of aminotransferases. Streptococci appeared to become abundant in the late stage of liver disease and were associated with a major risk of hyperammonaemia, while *S. salivarius* was especially increased in HCC patients [38].

In contrast with the previously published literature, Sultan et al. demonstrated an increased gut microbiota diversity in treatment-naïve patients with HCV infection compared to healthy controls, with a higher abundance of *Prevotella*, *Succinivibrio*, *Collinsella*, *Faecalibacterium*, *Coriobacteriaceae*, *Catenibacterium*, *Megasphera, Mitsuokella multacida*, and Ruminococcaceae, and a lower abundance of *Bacteroides, Dialister, Alistipes, Bilophila, Streptococcus,* and Enterobacteriaceae [39]. 

In patients with HCV-related liver cirrhosis, Bajaj et al. reported that treatment with pegylated interferon and ribavirin achieved a sustained viral response (SVR), but did not improve gut microbiota composition, systemic inflammation, or endotoxemia [40]. Conversely, in another study, treatment with direct acting antivirals (DAAs) was able to modify the composition of the gut microbiota at least one year after the achievement of SVR, reducing gut dysbiosis and improving systemic inflammation, but without any effect on intestinal permeability [41]. More recently, Welloner et al. showed that alpha diversity and the relative abundance of *Collinsella* spp. and *Bifidobacterium* spp. increased significantly 6–12 months after the achievement of SVR only in non-cirrhotic patients [42]. This confirms that treatment with DAAs can improve the gut microbiota profile in the short-term, especially in the early stage of liver disease, while in cirrhotic patients restoration of the gut microbiota may require more time.

### 3.3. Alcoholic Hepatitis

Alcohol is a well-known disruptor of the intestinal barrier; acting on its components, such as mucus and tight junctions, it favors bacterial translocation as well as inflammation [11].

Patients with alcohol use disorder (AUD) without alcoholic liver disease show quantitative and qualitative alterations of the gut microbiota. Indeed, a higher prevalence of bacterial overgrowth has been described [43], and the composition of the gut-microbiota is characterized by the reduction of beneficial bacteria, such as *Lactobacillus*, *Bifidobacterium* and Ruminococcaceae, and the increase in potentially pathogenic ones, such as Veillonellaceae and Enterobacteriaceae [44,45]. Llopis et al. showed that patients with severe alcoholic hepatitis had more Bifidobacteria and Streptococci than AUD patients without alcoholic hepatitis. Moreover, Enterobacteria and Streptococci were positively correlated with alcoholic hepatitis scores, and Enterobacteria also correlated with serum bilirubin levels [46]. The dysbiosis due to alcohol abuse contributes to the occurrence and progression of alcoholic liver disease also through metabolic effects, such as modulating GABA and energy metabolism [47].

FMT has been mainly studied in the setting of alcoholic hepatitis (Table 1). Intriguingly, germ-free mice receiving the gut microbiota from a patient with severe alcoholic hepatitis developed more severe liver inflammation, higher liver necrosis, increased intestinal permeability, and bacterial translocation than mice receiving the gut microbiota from AUD patients without alcoholic hepatitis [46].

In a pilot study, 8 male patients with severe alcoholic hepatitis ineligible for steroid treatment underwent FMT through a nasoduodenal tube daily for 7 days (Table 1) [25]. FMT was safe and the bilirubin Child–Turcotte–Pugh score model for end-stage liver disease (MELD) improved significantly within the first week after FMT compared to a historical cohort of 18 patients with severe alcoholic hepatitis treated with the SOC during the same period. The 1-year survival rate was significantly better in the FMT group compared to the historical controls. The analysis of the gut microbiota showed changes in relative abundance of pathogenic species, such as *Klebsiella pneumoniae* (relative abundance change: from 10% to <1% at 1 year), and nonpathogenic ones, such as *Enterococcus villorum* (relative abundance change: from 9% to 23% at 6 months), *Bifidobacterium longum* (relative abundance change: from 6% to 50% at 6 months), and *Megasphaera elsdenii* (relative abundance change: from 10% to 60% at 1 year).

A retrospective study compared the outcomes of patients with severe alcoholic hepatitis treated with nutritional therapy (17), corticosteroids (8), pentoxifylline (10), or FMT (16) (Table 1) [26]. Patients undergoing FMT experienced a significantly higher 90-day survival compared to the other groups, with a durable beneficial modulation of the gut microbiota.

Based on these data, FMT could be a promising strategy to treat severe alcoholic hepatitis, however caution is required due to the small simple size of the available studiesand the lack of randomized trials.

### 3.4. Nonalcoholic Fatty Liver Disease

Nonalcoholic fatty liver disease (NAFLD), recently renamed metabolic associated fatty liver disease (MAFLD) [48], has recently become the leading cause of chronic liver disease in Western countries [49]. The gut microbiota is involved in the pathogenesis and progression of MAFLD through metabolic and inflammatory pathways [50]. In particular, *Bacteroides* and *Ruminococcus* abundance correlate with the severity of liver inflammation and fibrosis [51]. In children with biopsy-proven nonalcoholic steatohepatitis (NASH) compared to healthy ones, a statistically significant increase in Bacteroidetes, Proteobacteria, Enterobacteriaceae and *Escherichia* has been reported, together with a decrease in Firmicutes [52]. Firmicutes can produce butyrate, which is a source of energy for enterocytes and helps to preserve gut permeability and avoid systemic inflammation [53]. Moreover, ethanol-producing bacteria (e.g. *Escherichia coli, Klebsiella spp.*) are overabundant in patients with NASH, who have significantly elevated blood ethanol concentrations when compared with healthy controls [52]. This can worsen liver inflammation and steatohepatitis, especially in the case of higher sugar consumption, leading to the so called “auto-brewery syndrome” [54].

Specific gut microbiome signatures (e.g. reduction in Firmicutes and increase in *Escherichia coli* and Proteobacteria) able to predict the presence of advanced fibrosis have been used in patients with NAFLD [55]. More recently, Loomba et al. identified a gut microbiome signature able to predict the presence of NAFLD-cirrhosis with high accuracy (area under the curve (AUC) of 0.91). This model was based on 19 discriminatory species, 12 increased (*Escherichia coli, Veillonella parvula, Veillonella atypica, Ruminococcus gravus, Clostridium boltae,* and *Acidaminococcus sp. D21*) and 7 reduced (*Eubacterium eligens, Eubacterium rectale,* and *Faecalibacterium prausnitzii*)*. Veillonella* and *Faecalibacterium* were the species with the highest discriminatory value. The model was effective in the prediction of cirrhosis independently of environmental and genetics factors, especially when incorporating clinical and laboratory parameters [56]. 

The alteration of the gut microbiota in cirrhotic patients with MAFLD has also been correlated with the development of HCC [57,58]. In particular, the reduction in beneficial bacteria such as *Akkermansia* and *Bifidobacterium* and the increase in *Bacteroides* was linked with the intestinal and systemic inflammatory profile. The consequent persistent stimulation of immune cells can favor the process of hepatocarcinogenesis through the exhaustion of the immune response, causing a kind of “immune paralysis”. This mechanism could be particularly true for the initiation of tumorigenesis, and may subsequently act as a trigger for the promotion of tumor cell proliferation, acting on the tumor microenvironment [59]. Another recent study investigated the correlation between gut microbiota signature, metabolomic profile and immune status in patient with MAFLD-HCC compared with MAFLD-cirrhosis. In MAFLD-HCC, dysbiosis was characterized by reduced alpha-diversity, with enrichment of some species, particularly *Bacteroides caecimuris* and *Veillonella parvula,* compared to MAFLD-cirrhosis. This signature was positively correlated with the production of short chain fatty acids (SCFAs), which exert immunomodulatory effects through the expansion of regulatory T cells and the decrease of CD^8+^ T cells [60].

There are only a few studies on the efficacy of FMT in the context of NAFLD (Table 1). In mice models of high-fat diet-induced steatohepatitis, FMT was effective in the modulation of the gut microbiota favoring the overgrowth of beneficial taxa [61]. The same study showed that FMT could improve tight junctions, decrease endotoxin serum levels, and ameliorate steatohepatitis, leading to a significant decrease in intrahepatic lipid accumulation, intrahepatic pro-inflammatory cytokines, and improving the NAS score. In humans, a recent study analyzed the efficacy of FMT in patients with NAFLD in terms of a decrease in insulin resistance, measured with HOMA-IR score, reduction of liver fat content, evaluated measuring hepatic proton density fat fraction (PDFF) by magnetic resonance imaging (MRI), and improvement in small intestinal permeability assessed using the lactulose/mannitol test [27]. After FMT, there was no significant improvement in insulin sensitivity or hepatic PDFF, but only in small intestinal permeability.

This evidence suggests that FMT may provide therapeutic benefit in NAFLD, and at least three clinical trials examining FMT in adults with biopsy-confirmed NASH are actively recruiting subjects (Clinicaltrials.gov, NCT02469272, NCT03803540, NCT02721264).

### 3.5. Primary Sclerosing Cholangitis, Primary Biliary Cholangitis and Autoimmune Liver Disease

The pathogenesis of primary sclerosing cholangitis (PSC) is still largely unclear [62]. Although genetic and environmental factors have been advocated, the association with inflammatory bowel diseases (IBD) in up to 70% of cases suggests a crucial role of the gut-liver axis. Gut microbiota composition and metabolic function, as well as the systemic and local immune response related to gut barrier permeability and bacterial translocation, can contribute to cholangiocytes injury, inflammation, and progression of fibrosis. Moreover, studies in animal models have demonstrated that enteric dysbiosis can lead to PSC-like alterations in the liver [63].

A specific gut microbiota signature allows us to discriminate PSC patients from healthy controls, independently of the association with IBD [64]. PSC patients show a reduced bacterial diversity and three bacterial genera, i.e. *Enterococcus, Fusobacterium*, and *Lactobacillus,* have been found to be significantly over-represented in PSC patients compared with healthy controls, independently of the severity of liver fibrosis, a previous history of liver transplantation, the concomitant presence of IBD, or ongoing treatment with ursodeoxycholic acid (UDCA) [64]. These changes in the gut microbiota composition could represent an important trigger for immune dysregulation. Indeed, gut-homing cells, T lymphocytes normally located in the lamina propria of the small intestine, and characterized by the expression of specific adhesion molecules, such as CCL25 and MadCAM-1, have been found in explant livers from patients with PSC [65]. This finding can be explained by the expression of adhesion molecules also in the hepatic sinusoids, which can favor the recruitment of gut-homing T cells in patients with PSC and contribute to the progression of biliary damage.

Interesting data have opened the field to the use of FMT in PSC patients (Table 1). An open-label pilot study evaluated the safety and efficacy (defined as a decrease in alkaline phosphatase [ALP] serum levels > 50%) of FMT in patients with PSC and concurrent IBD (Table 1) [28]. All 10 patients included in the study received FMT from a single donor by colonoscopy without the administration of antibiotics before the procedure. There were no FMT-related adverse events, and the intention-to-treat analysis showed a > 50% decrease in ALP serum levels in 30% of patients. The analysis of stool samples highlighted that FMT was able to increase bacterial diversity and the strength of the bacterial engraftment correlated with the decrease in ALP levels.

Since enteric but not colonic dysbiosis leads to hepatobiliary inflammation in PSC animal models, the upper small bowel could represent the ideal route for FMT in patients with PSC, as successfully demonstrated in a recent case report (Table 1) [29]. Hopefully, future investigations will further clarify this point.

Alterations of the gut microbiota have also been recognized in patients with primary biliary cholangitis (PBC). However, whether these alterations are the cause or consequence of liver disease is still unknown. Gut microbiota contributes to maintaining immune homeostasis, so it has been supposed that dysbiosis can trigger hepatic immune dysregulation through different mechanisms, such as cross-reactivity and molecular mimicry [66]. Furthermore, gut microbiota plays a pivotal role in the regulation of enterohepatic circulation of bile acids. Recent evidence underlines the association between dysbiosis and altered serum and fecal bile acid profile in PBC patients [67]. 

Tang et al. analyzed the difference between the gut microbiota of 60 patients with treatment naive PBC compared to 80 healthy controls, and then evaluated the modifications after treatment with UDCA. Treatment naive PBC patients had a reduced gut microbiota alpha-diversity compared with healthy controls, a decreased abundance of Bacteroidetes, and an increase in Fusobacteria and Proteobacteria. Eight genera were significantly increased in PBC patients, including *Haemophilus, Veillonella, Clostridium, Lactobacillus, Streptococcus, Pseudomonas, Klebsiella*, and an unknown genus of Enterobacteriaceae; conversely, *Sutterella, Oscillospira*, and *Faecalibacterium* were decreased compared with controls. Interestingly, the evaluation of the gut microbiota profile after treatment with UDCA showed a reduction of *Haemophilus* spp., *Streptococcus* spp., and *Pseudomonas* spp., and an increase of bacterial genera that were enriched in controls (*Bacteroides*, *Sutterella* spp. and *Oscillospira* spp.) [68]. 

According to the actual knowledge, the immunological alterations associated with the pathogenesis of autoimmune hepatitis (AIH) are triggered by the interaction between genetic predisposition and environmental factors. In a recent study, Wei et al. analyzed the gut microbiota composition in steroid-naïve AIH compared to healthy controls [69]. The results demonstrated a decrease in gut microbiota diversity and an increased abundance of *Veillonella*, which was strongly associated with the severity of liver inflammation. The authors also built a model able to distinguish patients with AIH from healthy controls based on the gut microbiota composition, in particular on the combination of *Veillonella*, *Lactobacillus*, *Oscillospira*, and Clostridiales.

At present, no study has evaluated the efficacy and safety of FMT in patients with PBC or AIH.

## 4. Future Perspectives

The gut microbiota plays a crucial role in the modulation of the immune system and the inflammatory response, and is also involved in the regulation of several metabolic pathways. In the last few years, several studies have shown a link between chronic liver disease and the gut microbiota. Even though our understanding of the interaction between the gut and the liver is still incomplete, dysbiosis seems to be the mainstay of chronic liver disease at any stage. The dynamic changes of the gut microbiota play an important role in the initiation and progression of liver injury, and probably represent the first hit in specific settings, such as NAFLD.

In light of this, the characterization of the gut microbiota and its modulation are promising diagnostic, prognostic, and therapeutic tools. Microbial signatures used in addition to clinical parameters can help in early diagnosis and in the avoidance of invasive diagnostic procedures. A shift in the gut microbiota composition during patients’ follow-up or during the course of hospitalization could help to identify patients at high risk of unfavorable clinical evolution. Nevertheless, targeted approaches in the modulation of the gut microbiota may be more effective than the use of broad-spectrum antibiotics or conventional probiotics. In this regard, FMT is the most powerful tool to reset the gut microbiota derangement consequent to chronic liver disease, and should be implemented in daily clinical practice to act synergistically with conventional medical therapy.

At present, there are no standardized guidelines regarding the indications and the choice of the ideal FMT procedure to be used in clinical practice in patients with chronic liver disease. In the previously analyzed studies, FMT was administered with different schedules as regards dosing and frequency, delivery modalities, and formulation. Although endoscopic techniques are reasonably safe, they are costly, invasive, they have potential procedural risks, and their repeatability over time may be limited. Enemas and nasoduodenal tubes are less invasive and less expensive but have some limitations. Enemas can fail to deliver fecal material throughout the colon if retention of the instilled material is not adequate. Nasoduodenal tube placement could elicit nausea, regurgitation, vomiting, and aspiration. The administration of FMT through oral capsules appear the safest and less expensive technique to deliver bacteria to the intestine. However further studies are needed to identify the ideal method of FMT administration in patients with chronic liver disease.

## Figures and Tables

**Table 1:** Evidence on fecal microbiota transplantation (FMT) in patients with chronic liver disease.

	Setting	Study Design	Groups	Type of FMT	Safety Outcomes	Efficacy Outcomes
Bajaj et al., (2017 and 2019) [20,21]	Recurrent HE in cirrhosis	Open-label, randomized clinical	FMT + SOC (10) vs. SOC (10)	Three FMT units instilled by enema	Patient with SAEs: 2 vs. 8 (*p* = 0.02) Total SAEs: 2 vs. 11 (*p* = 0.01)	Total HE episodes: 0 vs. 6 (*p* = 0.03) Improvement in PHES total score: - In FMT group (*p* = 0.03) - In SOC group (*p* = 0.98) Improvement in EncephalApp Stroop: - In FMT group (*p* = 0.01) In SOC group (*p* = 0.26) Long-term impact of FMT (12–15 months): - Hospitalization: 1 vs. 10 (*p* = 0.05) - Total HE events: 0 vs. 8 (*p* = 0.03)
Bajaj et al., (2019) [22]	Recurrent HE in cirrhosis	Randomized, single-blind, placebo-controlled	FMT + SOC (10) vs. placebo + SOC (10)	15 FMT capsules	Patient with SAEs: 1 vs. 6 Total SAEs: 1 vs. 11 Patient with self-limited AEs: 4 vs. 3	Total HE episodes: 1 vs. 7 HE episodes requiring hospitalization/ER visit: 1 vs. 7
Ren et al., (2017) [23]	HBeAg-positive CHB	Pilot study	AVT + FMT (5) vs. AVT (13)	FMT in duodenum, every 4 weeks until HBeAg clearance was achieved (one to seven FMT)	Adverse events: 0/5	HBeAg clearance: 4/5 vs. 0/13 (*p* = 0.001) HBsAg seroconversion: 0/5 vs. 0/13
Chauhan et al., (2020) [24]	HBeAg-positive CHB	Pilot study	AVT + FMT (14) vs. AVT (15)	Six cycles of endoscopic FMT in duodenum, every four weeks (12/14 complete cycles)	Minor adverse events: 6/14	HBeAg clearance: 2/12 vs. 0/15 (*p* = 0.188) HBsAg loss: 0/14 vs. 0/15 HBV-DNA suppression: 1/4 vs. 0/2
Philips et al., (2017) [25]	Severe AH	Pilot study	Steroids not eligible + FMT (8) vs. SOC (18)	Once daily FMT via nasoduodenal tube for 7 days		1-year survival: 87.5% vs. 33.3% (*p* = 0.018)
Philips et al., (2018) [26]	Severe AH	Retrospective	FMT (16) vs. corticosteroids (8), nutritional therapy (17) and, pentoxifylline (10)	Once daily FMT via nasoduodenal tube for 7 days		Survival in FMT vs. corticosteroids, nutritional therapy and pentoxifylline group: 30-day survival: 75% vs. 63, 47, and 40% (*p* = 0.179) 90-day survival: 75% vs. 29, 38 and 30% (*p* = 0.036)
Craven et al., (2020) [27]	NAFLD	Double-blinded randomized controlled	Allogenic FMT (15) vs. autologous FMT (6)	Single endoscopic FMT in duodenum		HOMA-IR score after 6 weeks: no significant decrease Hepatic PDFF after 6 months: no significant changes Small intestinal permeability after 6 weeks: improvement in allogenic group (*p* = 0.018)
Allegretti et al., (2019) [28]	PSC and concurrent IBD	Open-label pilot study	FMT (10)	Single endoscopic FMT in right colon	FMT-related AEs: 0/10	Decrease in ALP levels ≥ 50%: 3/10
Philips et al., (2018) [29]	PSC without IBD	Case report	-	Endoscopic FMT in duodenum, Once weekly for 4 weeks		

SOC = standard of care, HE = hepatic encephalopathy, SAEs = serious adverse events, AEs = adverse events, PHES = psychometric encephalopathy score, AH = alcoholic hepatis, ER = emergency room, NAFLD = non-alcoholic fatty liver disease, HOMA-IR = homeostatic model assessment for insulin resistance, PDFF = proton density fat fraction, PSC = primary sclerosing cholangitis, IBD = inflammatory bowel disease, ALP = alkaline phosphatase, CHB = chronic B hepatitis, AVT = antiviral therapy.

## Data Availability

Not applicable.
